# Treatment preferences as basis for decision making in patients using direct oral anticoagulants in Spain

**DOI:** 10.1007/s11239-020-02194-5

**Published:** 2020-06-27

**Authors:** Olga Gavín, Jesús Grandes, Ma Almudena García, Cristina Marzo, Alejandro Curcio, Rosa Arístegui, Marta González, Juan José Cerezo-Manchado

**Affiliations:** 1 Hematology and Hemotherapy Service, Hospital Clínico Universitario Lozano Blesa, Avda. San Juan Bosco, 15, 50009 Zaragoza, Spain; 2 Internal Medicine Service, Complexo Hospitalario Universitario de Vigo, Vigo, Spain; 3 Hematology and Hemotherapy Unit, Hospital Universitario Virgen de las Nieves, Granada, Spain; 4 Haematology Service, Hospital Universitari Arnau de Vilanova, Lleida, Spain; 5 Cardiology Service, Hospital Universitario de Fuenlabrada, Madrid, Spain; 6 Daiichi Sankyo España, Madrid, Spain; 7 Haematology Service, Hospital Universitario Santa Lucia, Cartagena, Spain

**Keywords:** Atrial fibrillation, Anticoagulant drugs, Patient preference, Selection for treatment

## Abstract

Treatment preferences are considered a relevant decision-making driver by the main atrial fibrillation (AF) guidelines. Direct Oral Anticoagulants (DOACs), considered as similar clinically, have administration differences useful for treatment individualization. Preferences, priorities and satisfaction of DOAC users were assessed through an observational, multicentric (25 hospitals), cross-sectional study including adult AF-patients (and/or caregivers) in Spain. Three study groups were considered according to DOAC posology preferences: (A) once-daily, with water; (B) once-daily, with food; (C) twice-daily. Overall, 332 patients and 55 caregivers were included. Mean (SD) age was 73.7 (10.7) years [58.7 (13.9) for caregivers]; 51.5% women [69.1% for caregivers]; 80.7% showed comorbidities and poly-pharmacy [6.6 (3.3) drugs/day]. No statistically significant differences were shown among study groups. Once-daily administration was preferred by 274 patients (82.5%) [60.8% (Group A); 21.7% (Group B); 17.5% (Group C)], and 47 caregivers (85.5%) [58.2% (Group A); 27.3% (Group B); 14.5% (Group C)]. Once-daily DOACs were prescribed in 42.8% of the patients. Bleeding risk was the main concern for both, patients and caregivers, followed by DOAC posology and interactions. Although treatment satisfaction (patients and caregivers) was high (9.0 and 9.1 points, respectively), match between individual treatment preferences and real prescriptions was only shown in 41.0% of AF-patients, evidencing a need for patient involvement on treatment decision-making. There is not a patient profile linked to treatment preferences, and clinical criteria must be the main driver for decision-making. However, for most AF-patients (elderly patients), aged, with comorbidity, poly-pharmacy and high cardiovascular risk, once-daily DOACs would be the preferred option.

**Electronic supplementary material:**

The online version of this article (10.1007/s11239-020-02194-5) contains supplementary material, which is available to authorized users.

## Highlights

- Once-daily administration is the preferred option for anticoagulation treatment, according to the self-reported preference of 82.5% of AF patients and 85.5% of their caregivers. However, once-daily DOACs are prescribed in less than the half of the AF patients (42.8%).
- Considering the individual preferences of each patient and the prescribed DOAC, coincidence would be observed only in 41.0% of the study patients.
- Bleeding risk was the most important attribute regarding DOAC therapy from patients and caregivers’ perspective (7.3 and 8.0 points over 10, respectively). The number of daily administrations was ranked at the same importance level that minor bleeding risk by the patients (5.6 points) and at drug-drug interactions level by the caregivers (6.3 points), resulting in a relevant topic to be considered for decision-making.
- Treatment preferences, regarding DOAC, were not linked to specific patient profiles. However, a common patient profile was observed for DOAC users (patient with comorbidities, poly-pharmacy, low physical activity and moderate/high cardiovascular risk), that could be described as “elderly patient”. For these patients, the most probably preferred therapy would be a once-daily dose DOAC.
- DOAC selection must be conducted according to clinical criteria by the main prescriber. However, considering patients and caregivers priorities, when different options are available, final decision-making should include patient and/or caregiver preferences, being necessary to include this parameter in the routine clinical practice.

## Introduction

Atrial fibrillation (AF) is the most frequent arrhythmia, affecting around 5% of the population older than 65 years (18% for ≥85 years) [1, 2], and one of the main stroke factor risks [3]. Traditionally, vitamin K antagonists (VKA) were the main drugs used to prevent AF-related stroke and systemic embolism [4, 5]. Although VKAs have proven to be effective, they are also characterized by well-known limitations such as: narrow therapeutic window, low predictable response, relevant drug and food interactions, slow start and end of action, need for periodic controls and dose adjustments [6, 7]. Moreover, around 40% of AF-patients receiving VKAs in Primary Care have poor anticoagulation control in Spain [8]. In this context, Direct Oral Anticoagulants (DOACs) have been adopted as useful alternatives to VKAs in routine clinical practice [8–10], being at least as effective as warfarin in the prevention of AF-related stroke and systemic embolism, with a better safety profile, particularly regarding intracranial haemorrhage risk [10–14].

Currently, four DOACs are marketed in Spain [15]: edoxaban, apixaban, rivaroxaban and dabigatran, all of them with a similar pharmacological profile and indication [15, 16]. DOAC selection is mainly conducted according to the clinical experience of the prescriber, on a basis of cardiovascular risk, patient comorbidities and potential interactions [17], as non-conclusive studies have been conducted for assessing differences among DOACs and no recommendations or guidelines are available to position these DOACs according to potential patient profiles [4, 17–19]. Despite that, DOACs have intrinsic differences that could be used as drivers for drug selection, mainly based on their different posology and administration characteristics [15, 16].

Most healthcare systems agree on the importance of involving the patient in healthcare decision-making, not only in terms of disease awareness, but on treatment selection, as treatment adherence is crucial for outcomes achievement [20]. However, most AF patients could be aged, and a direct involvement for treatment decision-making with focus on direct anticoagulation could be difficult, being necessary, the potential involvement of the caregiver or the consideration of added factors such as poly-pharmacy.

It is clear that effectiveness and safety are the key parameters in the selection of the most appropriate drug for both, patients and prescribers [21–23]. However, beyond the clinical criteria, the need to succeed in the most accurate treatment administration is the priority [16, 17], being necessary, on one hand, the involvement of the patient on treatment selection [24], and, on the other hand, when the patient could not be actively involved, identifying the most appropriated therapy according to the patient profile.

In this regard, previous studies suggested that differential attributes of the available DOACs could be used as drivers for treatment decision-making, being potentially linked to patient profiles and preferences [21, 25]. According to that, the main objective of our study was to identify patient (and caregiver) preferences and attributes importance regarding DOACs administration, as well as their potential link with patient/caregiver profiles. The final goal of this information was aimed to contribute to an improvement of the treatment individualization and shared decision-making regarding anticoagulation in routine clinical practice.

## Methods

### Study design and population

Observational, non-interventional, cross-sectional and multicentre study, involving 25 hospital centres in Spain. Patients included had ≥18 years, diagnosed with AF and treated with a DOAC for ≥6 months according to routine clinical practice in Spain. When available during the study visit, patient’s caregiver was also invited to participate. The included patients, and /or their caregivers, had to be able to understand and respond autonomously to the study questionnaire.

Patients and/or caregivers included in the study were classified in three study arms depending on their main treatment preference regarding DOAC posology (self-reported): Group A (once-daily dose with water), Group B (once-daily dose with food) and Group C (twice-daily dose).

### Study variables

Main study variables were collected from patient’s medical history and from a study questionnaire ad-hoc designed, and validated by six clinical experts on anticoagulation management in Spain (supplementary materials) [25]. Importance, prevalence and satisfaction questions were based in 10-points scoring ranges.

Sociodemographic data, including gender, age, work situation, personal situation and life style variables were collected for patients and caregivers. Clinical data, including: comorbidities, cardiovascular risk, DOAC treatment, poly-pharmacy, drug interactions knowledge, administration capabilities and prescriber profile were collected from patients. Treatment preferences and importance of bleeding, interactions, posology and administration needs, were assessed for patients and caregivers.

All study data were included in an electronic Clinical Research Form (eCRF) specifically designed for the study and managed through a web application ensuring the anonymity, confidentiality and safety of the reported data. All the study variables were referred to routine clinical practice.

### Sample size estimation

Since there are no specific data on DOAC treatment preference in Spain, in order to identify the preferences of patients and / or their caregivers for one of the 3 treatment options considered (A: once-daily dose with water; B: once-daily dose with food; C: twice-daily dose), 336 patients on active DOAC treatment was considered as needed. This sample size would be the needed to identify the patients’ preference for a certain option (Option A, B or C) with an accuracy <5.5% and an alpha significance level of 0.05, assuming the maximum indeterminacy criterion. If the patient cannot answer, the caregiver’s preference will be considered, when available.

Regarding caregivers, it was estimated that approximately 51% of DOAC- treated patients should have a caregiver [25], which makes a sample of 172 subjects sufficient to estimate preferences for a caregivers’ treatment option, with an accuracy of 7.5%.

### Data analysis

Statistical analysis was carried out in all the evaluable patients. Descriptive analyses of all variables were conducted separately, using (i) absolute and relative frequencies for discrete variables (qualitative and quantitative), and (ii) average statistics, standard deviation (SD), extreme values and quartiles, for continuous quantitative variables. For all comparisons a statistical significance level of 0.05 was considered. Statistical package SAS (Statistical Analysis System) version 9.2 or later for Windows was used.

The target sample for the study was estimated in 336 patients (caregivers were included according to the routine clinical practice, with no target sample pre-defined). Data analyses were performed considering patients’ distribution according to their treatment preferences (Group A, B and C). The study variables were analysed comparatively according to these 3 study arms.

### Ethics considerations

The study agreed the ethical principles of the Declaration of Helsinki and the guidelines specified in Order SAS / 3470/2009 of the *Agencia Española de Medicamentos y Productos Sanitarios* (AEMPS). The study was classified as EPA-OD (Post-authorisation Study, Other designs) by the AEMPS and evaluated and approved by the reference Clinical Research Ethics Committee (CEIm) of the Hospital de Fuenlabrada (Madrid), as well as the CEIms of the participating hospitals, as needed.

All the patients and caregivers were informed by the investigators about the study purposes and signed the informed consent prior to study inclusion.

## Results

A total of 335 patients and 55 caregivers were recruited, being considered as valid for data analysis 332 patients (99.1%) and all the caregivers. The study questionnaire was answered autonomously by the patient (83.4% of the cases), together with the caregiver (11.1%, each one according to his/her criteria) or only by the caregiver (5.4%).

According to the self-reported preferences, 60.8% of DOAC users would prefer a once-daily administration with water (Group A), 21.7% a once-daily administration with food (Group B) and 17.5% twice-daily administrations (Group C). Considering caregivers’ preferences, 58.2% were included in Group A, 27.3% in Group B and 14.5% in Group C.

### Socio-demographic profile of patients and caregivers

Patients included in the study (DOAC users) showed a mean age (SD) of 73.7 (10.7) years and 51.5% were males (Table 1). Around the half of the included patients reported to be supported for a caregiver (45.2%), being in most cases informal (84.7%), even though only 16.6% participated in the study (were present during the study visit). The socio-demographic profile of the participant caregivers is shown in Table 2. No statistically significant differences were shown between study groups that could indicate different patient profiles (socio-demographic parameters) related to treatment preferences (Tables 1 and 2).

**Table 1:** Socio-demographic and clinical characteristics of the study patients

	Group A	Group B	Group C	Total	P
	(*n* = 202)	(*n* = 72)	(*n* = 58)	(*n* = 332)	
Socio-demographic variables					
Gender (male); n (%)	104 (51.5)	36 (50.0)	31 (53.4)	171 (51.5)	0.9263
Age (years); mean (SD)	73.9 (11.0)	74.9 (9.9)	71.4 (10.6)	73.7 (10.7)	0.1509
Personal situation; n (%)					
Lives alone, autonomously	55 (27.2)	15 (20.8)	18 (31.0)	88 (26.5)	0.1103
Lives alone, with support	2 (1.0)	3 (4.2)	1 (1.7)	6 (1.8)	
Lives accompanied, spending most of time alone	23 (11.4)	2 (2.8)	4 (6.9)	29 (8.7)	
Lives alone and mainly accompanied	122 (60.4)	52 (72.2)	35 (60.3)	209 (63.0)	
Physical activity; n (%)					
No exercise	68 (33.7)	20 (27.8)	15 (25.9)	103 (31.0)	0.2466
Occasional exercise	105 (52.0)	33 (45.8)	29 (50.0)	167 (50.3)	
Moderate exercise	16 (7.9)	11 (15.3)	6 (10.3)	33 (9.9)	
Regular exercise	13 (6.4)	8 (11.1)	8 (13.8)	29 (8.7)	
Working activity (active); n (%)	29 (14.4)	6 (8.3)	7 (12.1)	42 (12.7)	0.4139
Full time	14 (48.3)	1 (16.7)	3 (42.9)	18 (42.9)	0.7865
Intensive workday	8 (27.6)	2 (33.3)	1 (14.3)	11 (26.2)	
Part time	5 (17.2)	2 (33.3)	2 (28.6)	9 (21.4)	
Other	2 (6.9)	1 (16.7)	1 (14.3)	4 (9.5)	
Caring for a dependent person; n (%)	34 (16.8)	16 (22.2)	8 (13.8)	58 (17.5)	0.4213
Presence of caregiver; n (%)	95 (47.0)	33 (45.8)	22 (37.9)	150 (45.2)	0.4672
Formal / paid	14 (14.7)	7 (21.2)	2 (9.1)	23 (15.3)	0.4573
Informal /unpaid	81 (85.3)	26 (78.8)	20 (90.9)	127 (84.7)	
Clinical variables					
Presence of comorbidities; n (%)	159 (78.7)	62 (86.1)	47 (81.0)	268 (80.7)	0.3923
Renal failure	26 (12.9)	9 (12.5)	8 (13.8)	43 (13.0)	0.9750
Liver failure	1 (0.5)	1 (1.4)	0 (0.0)	2 (0.6)	0.5672
Cardiopathy	100 (49.5)	33 (45.8)	27 (46.6)	160 (48.2)	0.8343
Gastrointestinal disorders	14 (6.9)	9 (12.5)	5 (8.6)	28 (8.4)	0.3438
Neurological disease	27 (13.4)	8 (11.1)	8 (13.8)	43 (13.0)	0.8678
Other	66 (32.7)	36 (50.0)	26 (44.8)	128 (38.6)	0.0193
Cardiovascular risk; mean (SD)					
CHA_2_DS_2_-VASc scale	3.49 (1.33)	3.59 (1.29)	3.38 (1.41)	3.49 (1.34)	0.6632
HAS-BLED index	2.03 (1.09)	2.13 (1.08)	1.96 (1.03)	2.04 (1.07)	0.6710
Treatment variables					
Time treated with DOAC (months); mean (SD)	22.5 (16.5)	25.0 (20.1)	26.6 (20.5)	23.8 (18.1)	0.2577
Number of daily drugs administered; mean (SD)	6.5 (3.4)	6.6 (2.8)	7.0 (3.8)	6.6 (3.3)	0.5570
Administration moment; n (%)					
Morning	194 (96.0)	68 (94.4)	58 (100.0)	320 (96.4)	0.2206
Midday	106 (52.5)	41 (56.9)	26 (44.8)	173 (52.1)	0.3833
Evening	182 (90.1)	65 (90.3)	55 (94.8)	302 (91.0)	0.5277
Previous OAC used; n (%)					
None	90 (44.6)	33 (45.8)	23 (39.7)	146 (44.0)	0.7529
VKA	101 (50.0)	36 (50.0)	31 (53.4)	168 (50.6)	0.8602
Other DOAC	11 (5.4)	3 (4.2)	4 (6.9)	18 (5.4)	0.8602
DOAC currently used; n (%):					
Edoxaban	48 (23.8)	10 (13.9)	1 (1.7)	59 (17.8)	<0.0001
Apixaban	77 (38.1)	17 (23.6)	35 (60.3)	129 (38.9)	
Rivaroxaban	50 (24.8)	32 (44.4)	1 (1.7)	83 (25.0)	
Dabigatran	27 (13.4)	13 (18.1)	21 (36.2)	61 (18.4)	
DOAC dose received; n (%)					
Standard dose	147 (72.8)	53 (73.6)	48 (82.8)	248 (74.7)	<0.0001
Reduced dose	55 (27.2)	19 (26.4)	10 (17.2)	84 (25.3)	
DOAC prescriber; n (%)					
Cardiologist	99 (49.0)	35 (48.6)	26 (44.8)	160 (48.2)	0.7180
Haematologist	63 (31.2)	22 (30.6)	22 (37.9)	107 (32.2)	
Internal medicine	15 (7.4)	2 (2.8)	2 (3.4)	19 (5.7)	
Neurologist	17 (8.4)	8 (11.1)	6 (10.3)	31 (9.3)	
Family doctor	7 (3.5)	3 (4.2)	1 (1.7)	11 (3.3)	
Emergency	1 (0.5)	2 (2.8)	1 (1.7)	4 (1.2)	
Patient awareness about DOAC interactions; n (%)					
Full	37 (18.3)	9 (12.5)	9 (15.5)	55 (16.6)	0.5928
Partial	59 (29.2)	19 (26.4)	20 (34.5)	98 (29.5)	
None	106 (52.5)	44 (61.1)	29 (50.0)	179 (53.9)	

*DOAC* Direct Oral AntiCoagulant, *OAC* Oral AntiCoagulant, *SD* Standard Deviation

**Table 2:** Socio-demographic characteristics of the study caregivers

	Group A	Group B	Group C	Total	P
	(*n* = 32)	(*n* = 15)	(*n* = 8)	(*n* = 55)	
Gender (male); n (%)	9 (52.9)	5 (29.4)	3 (17.6)	17 (30.9)	0.8521
Age (years); mean (SD)	59.9 (13.0)	55.9 (17.5)	58.9 (10.7)	58.7 (13.9)	0.6581
Physical activity; n (%)					
No exercise	6 (18.8)	4 (6.7)	2 (25.0)	9 (16.4)	0.1677
Occasional exercise	20 (62.5)	7 (46.7)	3 (37.5)	30 (54.5)	
Moderate exercise	4 (12.5)	3 (20.0)	0 (0.0)	7 (12.7)	
Regular exercise	2 (6.3)	4 (26.7)	3 (37.5)	9 (16.4)	
Working activity (active); n (%)	15 (46.9)	10 (66.7)	4 (50.0)	29 (52.7)	0.4420
Full time	7 (46.7)	2 (20.0)	1 (25.0)	10 (34.5)	0.4970
Intensive workday	5 (33.3)	5 (50.0)	3 (75.0)	13 (44.8)	
Part time	0 (0.0)	1 (10.0)	0 (0.0)	1 (3.4)	
Other	3 (20.0)	2 (20.0)	0 (0.0)	5 (17.2)	
Caring for other dependent persons; n (%)	15 (46.9)	5 (33.3)	4 (50.0)	24 (43.6)	0.6327
Caregiving period (month); mean (SD)	85.8 (110.0)	91.8 (172.9)	199.5 (185.9)	104.0 (144.1)	0.1261
Caregiving weekly time (h); mean (SD)	41.6 (41.3)	40.3 (57.3)	26.9 (32.8)	39.1 (44.7)	0.7076
Travel need for caregiving; n (%)	13 (40.6)	7 (46.7)	3 (37.5)	23 (41.8)	0.5337

*SD* Standard Deviation

### Clinical and therapeutic profile of AF patients treated with DOAC

Most AF-patients (80.7%) presented comorbidities, being the most frequent cardiopathies (48.2%) (Table 1). According to routine clinical practice, cardiovascular risk was measured through the CHA_2_DS_2_-VASc scale (98.8% of patients), and the HAS-BLED index (92.1%) (Table 1). DOAC users showed predominantly, a high cardiovascular risk, in most cases ≥2 according to CHA_2_DS_2_-VASc scale (95.0%). None of the clinical variables assessed showed impact on patient’s preferences.

Most of the participant patients (57.2%) had a twice-daily DOAC prescribed (apixaban or dabigatran), being the cardiologist and the haematologist the main prescribers for these drugs (Table 1). It should be noticed that 44% of the patients were naïve for oral anticoagulation. Most of the included patients showed poly-pharmacy, being treated with a mean (SD) of 6.6 (3.3) drugs daily, involving administrations at morning and evening. A total of 59.2% of patients reported no knowledge about DOACs interactions.

Regarding treatment variables (Table 1), the only item showing statistically significant differences among preference groups was the prescribed DOAC (drug and dose). Most of the patients that preferred Group B (once-daily dose with food), were rivaroxaban users (44.4%), and 96.6% of the patients selecting Group C (twice-daily dose drugs) were apixaban or dabigatran users. Group A (patients that would prefer once-daily dose, with water), included patients being treated according to routine clinical practice with, mainly, apixaban (38.1%) or rivaroxaban (24.8%), none of them coinciding with the patient’s self-reported preference (Table 1).

**Fig. 1:**
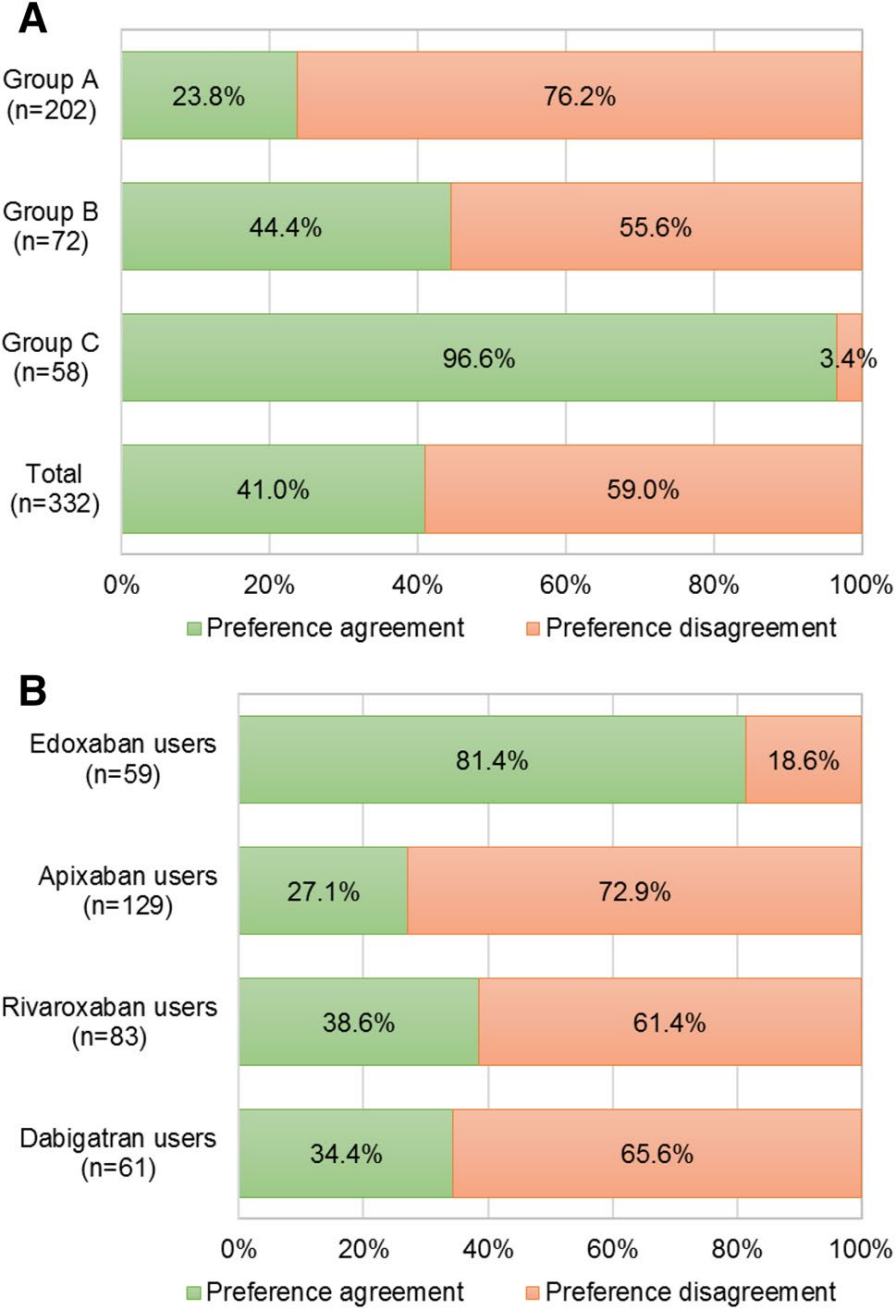
Level of agreement between patient reported preferences and DOAC prescribed, according to **a** preference group, and **b** DOAC prescribed

Considering the individual preferences and the prescribed DOAC, preference/prescription match was shown only in 41.0% of the cases (Fig. 1a). This percentage was reduced to 29.2% considering preference/prescription match in the once-daily groups (A and B). Regarding prescribed DOAC, the highest proportion of patients showing match between preference and prescription (81.4%) were those using edoxaban as anticoagulant drug (Fig. 1b).

### Patient’s and caregiver’s perception about DOAC treatment

**Fig. 2:**
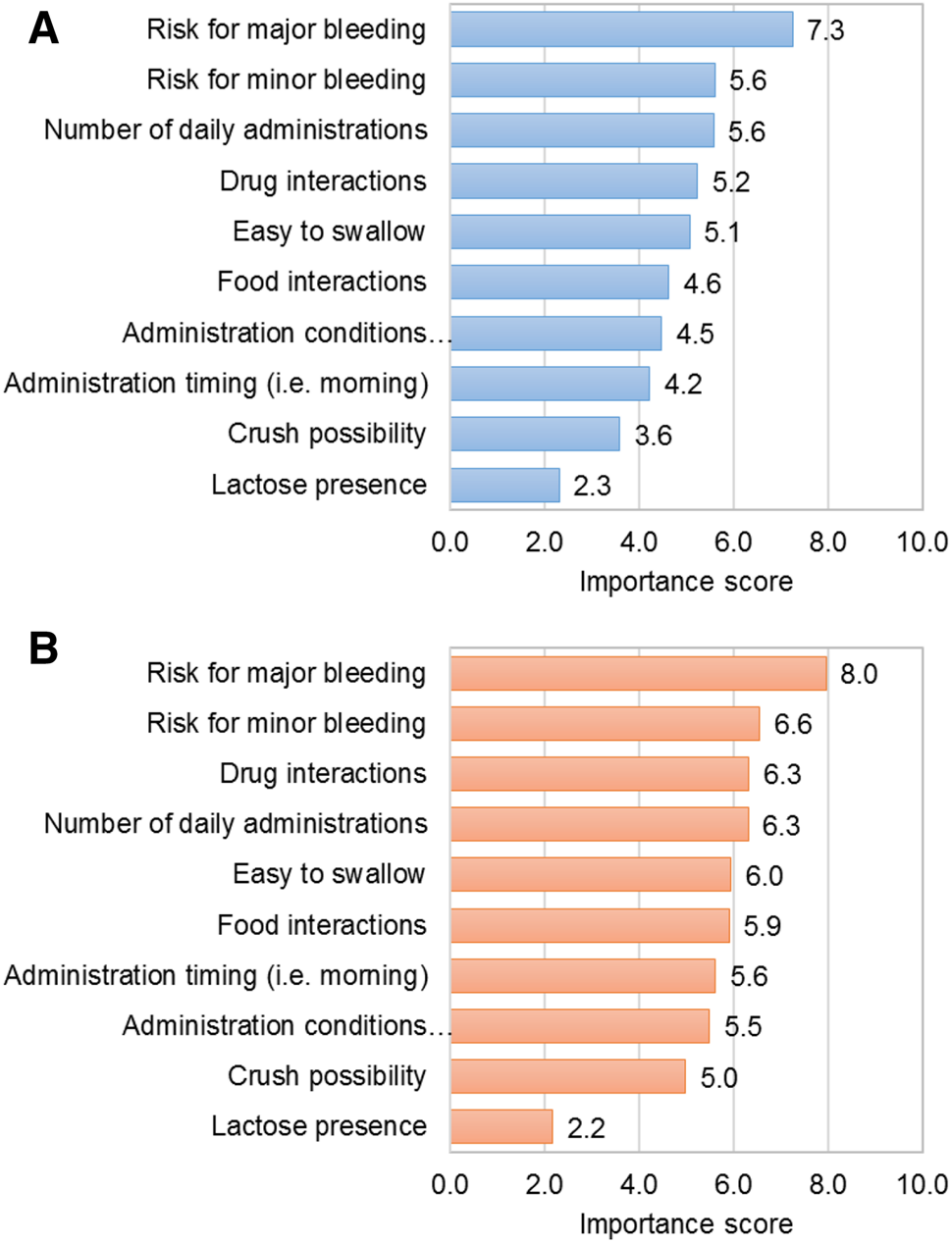
Importance value provided by **a** patients and **b** caregivers to the different attributes of DOACs

Both, patients and caregivers showed similar importance rates for the considered DOAC attributes, being major bleeding risk their common main priority (7.3 and 8.0 points over 10, respectively), followed by the concern for minor bleeding (5.6 and 6.6 points, respectively) (Fig. 2). The number of daily administrations was ranked on third place according to patients’ perspective (5.6 points), aligned with the importance provided to minor bleedings. From caregiver’s perspective, the importance of DOAC daily administrations was aligned with the importance provided to drug-drug interactions (6.3 points in both cases) (Fig. 2).

**Fig. 3:**
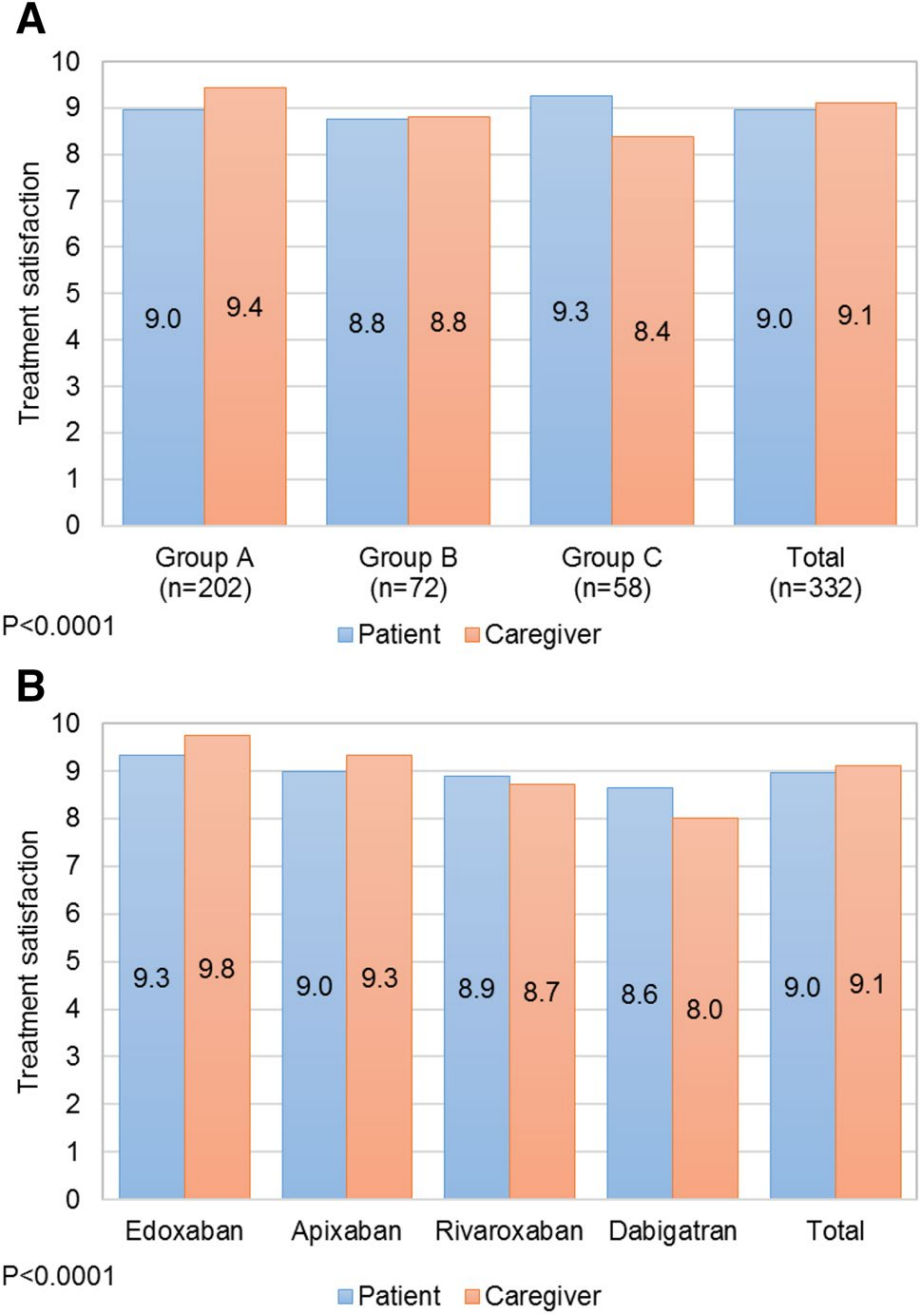
Satisfaction level with the DOAC therapy received reported by patients and caregivers according to **a** preference group **b** DOAC prescribed

Overall, treatment satisfaction reported by patients and caregivers was high [9.0 (1.4) and 9.1 (1.8) points, respectively], for all the preference groups (Fig. 3a). Results analysis per prescribed drug showed that edoxaban users were the most satisfied patients and caregivers [9.3 (0.9) and 9.8 (0.6), respectively] (Fig. 3b).

## Discussion

According to the main AF guidelines and recommendations [4], oral anticoagulation is a must for AF-patients, with the focus to prevent the majority of ischaemic strokes and prolong life, even in elderly patients, patients with cognitive dysfunction, or patients with frequent falls or frailty [4]. Drug selection could be conditioned by the patient profile, the clinical criteria, but also by the main clinical recommendations and guidelines [4, 5]. Specifically, DOACs should be the selection drugs used to prevent AF-related stroke and systemic embolism [4]. However, in Spain, DOACs use is highly conditioned by the Therapeutic Positioning Report [5], restricting the use of these drugs to well-defined patients, mainly, those that could not be appropriately controlled through VKAs.

It is known that around 40% of AF-patients would prefer to avoid VKA therapies due to the need for regular controls and interaction risk [23, 26]. In addition, several studies have been conducted for assessing patient preferences in terms of anticoagulation [22, 24, 26–28], all of them coinciding in the positioning for simple drug administrations. However, few studies have been conducted for addressing the ad-hoc preferences of patients and caregivers regarding DOACs [29].

In agreement with our results, bleeding-related risks are the main concerns for OAC users [21, 23–25, 27], placing administration characteristics as a secondary priority, by reinforcing that the clinical criteria is the prescription cornerstone. However, considering similar therapeutic options such as DOACs [16, 17], posology and treatment administration characteristics became relevant decision-making drivers, easy to be considered in routine clinical practice and contributing to an optimal drug selection [21, 29].

According to the preferences directly reported by the study participants, 82.5% of DOAC users and 85.5% of caregivers would prefer once-daily administrations, preferably with an intake independent of food [29]. However, only 42.8% of the patients were treated with a once-daily DOAC (rivaroxaban or edoxaban) according to the routine clinical practice in Spain. Considering the individual preferences of the patients and the prescription conducted, match was only evidenced in 41.0% of the cases. This data is fully aligned with the provided by a patient survey conducted in Spain, reporting a patient involvement on anticoagulation decision-making lower than 41% [24], and reinforcing the need for including the patient needs, together with the clinical criteria, for drug selection in routine clinical practice.

In Spain, the most preferred drug administration was once-daily, with water (for both, patients and caregivers), in agreement with the preferred option reported in similar European studies [29]. However, this was the study group with the highest disagreement between personal preferences and real prescriptions. The minority study group, including patients that preferred twice-dose administrations (Group C), was the group with the highest match between preference and prescription, evidencing a potential influence of the awareness tasks conducted by the prescribers in routine clinical practice, and the impact that could have on patient preferences [30, 31]. Treatment awareness is a must for a successful anticoagulation, even though it should be conducted also integrating the patient preferences, as far as possible, with no interference with the clinical criteria.

It seems clear that patients are being treated mainly according to clinical criteria and that prescribers are probably conducting an excellent awareness task about anticoagulation importance, as it has been shown by the high satisfaction level and concern about bleeding risk. However, they are not actively involving the patients or their caregivers in decision-making, and personal preferences are not being considered enough, in contrast to the recommended procedure included in the main AF guidelines [4].

Clearly, the main driver for drug selection must be effectiveness and safety, as well as potential contraindications for each patient. However, it has been widely demonstrated that treatment administration is also a very important criteria for both, patients and caregivers, being related with a successful compliance [7, 28]. It should be highlighted that AF is a chronic disease and that anticoagulation is a treatment to be considered for a long time, with a high cost for the National Healthcare System. In this regard, it is necessary to conduct an appropriated evaluation of the most suitable treatment to be prescribed to the patient by guaranteeing a good acceptability and compliance in order to achieve the best possible outcome results and the best possible public healthcare resource investment for each target patient.

The study results do not allow to develop a specific patient profile per DOAC. However, it could be defined a common patient profile with a main preference reported, that need to be ad-hoc assessed on routine clinical practice. In general terms, most of the study patients could be defined as elderly patients, being aged people, with associated comorbidities, low activity profile and multi-drug users. These patients mainly prefer the use of once-daily DOACs, with special emphasis for those administered with no need of food, in agreement to other European studies [29].

The present study was not exempt of limitations that should be considered for results interpretation. On one hand, main data was self-reported by patients and caregivers to the participant investigators, being a potential bias source, as they were directly interviewed for their prescribers (especially regarding treatment satisfaction scores). On the other hand, patients’ profile could be affected by the recruitment process, focusing on patients that could answer the study questionnaire. In this regard, the protocol asked for a consecutive recruitment of patients coming to the routine clinical visits, and in case of patients do not capable to answer the questions, the study provided the possibility for including the caregiver. Despite the study limitations, the collected results were coherent with the reported in similar studies, in both terms, patients profile [32, 33] and reported preferences [22, 24, 29], minimising the impact of the study limitations and providing robustness to the presented results.

## Conclusions

According to the main guidelines for AF management, patient preferences should be one of the cornerstones for anticoagulant decision-making, following the clinical criteria. However, patient preferences seem to be considered marginally in the routine clinical practice in Spain, showing a coincidence of 41% between treatment preferences and real prescriptions. Certainly, a full coincidence among patient preferences and the most appropriated prescription is not-affordable, as clinical decision-making is mandatory, and could differ from administration preferences. Nevertheless, in most cases, when clinical differences are not a selection factor, patient preferences should be the main driver, especially considering that they are a relevant concern for patients and caregivers, after bleeding risks.

The study results indicate that there are not specific patient profiles that could be pre-defined linked to patient preferences. According to the data collected from routine clinical practice in Spain, DOAC users would be aged patients, with associated comorbidities, low active physically and poly-medicated (elderly patients), and these patients would prefer once-daily DOACs, preferably not linked with food.

It is beyond discussion that clinical criteria must be the main driver for treatment decision-making and the main expert to conduct treatment choice is the prescriber. However, as patient preferences are not linked to pre-defined patient profiles, when different treatment options are possible, patient or caregiver preferences, assessed through a simple question should be considered. In this regard, specific guidelines or tools should be developed for helping in the balancing of clinical criteria and preferences for DOAC choice in routine clinical practice.

## Electronic supplementary material

Below is the link to the electronic supplementary material.

Electronic supplementary material 1 (DOCX 81 kb)

## Data Availability

Data and material could be ad-hoc requested to the study sponsor.
