# Supplementary material for: Treatment preferences as basis for decision making in patients using direct oral anticoagulants in Spain
Source: J Thromb Thrombolysis. 2020 Jun 27;51(2):475–84. doi: 10.1007/s11239-020-02194-5 (PMC7886773; doi:10.1007/s11239-020-02194-5)
Supplement: Supplementary file 1 — Electronic supplementary material 1 (DOCX 81 kb) [file 11239_2020_2194_MOESM1_ESM.docx]

**Treatment preferences for patient and/or caregiver**

**(Spanish; Spain)**

1. **Variables sociodemográficas del paciente**
   1. **Género del paciente:**

- Hombre
- Mujer
  1. **Edad del paciente:**

|  | Años |
| --- | --- |

- 1. **Estilo de vida del paciente:**
- **¿Cuál es la situación personal del paciente en su vivienda habitual?**
- Vive solo, de forma autónoma
- Vive solo, pero requiere de asistencia
- Vive acompañado, pero pasa la mayor parte del tiempo solo
- Vive y se encuentra acompañado la mayor parte del tiempo
- **¿Cuál de las siguientes posibilidades describe mejor la frecuencia con la que el paciente realiza actividad física en su tiempo libre?**
- No hago ejercicio. El tiempo libre lo ocupo de forma casi completamente sedentaria (leer, ver la televisión, etc.)
- Hago alguna actividad física o deportiva ocasional (caminar, pasear, ir en bicicleta, jardinería, gimnasia suave, actividades recreativas que requieren un ligero esfuerzo, etc.)
- Hago actividad física varias veces al mes (deportes, gimnasia, correr, natación, ciclismo, juegos de equipo, etc.)
- Hago entrenamiento deportivo o físico varias veces a la semana
- **¿En el momento actual, el paciente sigue alguna dieta en especial?**

| - Sí - No | - **¿Qué tipo de dieta?** - Vegetariana / Vegana - Hipocalórica - Hipercalórica - Hiposódica - Diabética - Baja en grasas - Otra (especificar): ___________________________ |
| --- | --- |

- **¿Tiene el paciente alguna intolerancia alimentaria?**

| - Sí - No - Desconocido | - **¿Qué tipo de intolerancia?** - Lactosa - Gluten - Otra (especificar): ___________________________ |
| --- | --- |

- **¿El paciente está activo laboralmente?:**

| - Sí - No | - **¿Qué tipo de jornada laboral realiza?** - Media Jornada - Jornada completa partida - Jornada intensiva (mañana o tarde) - Otra (especificar): ___________________________ |
| --- | --- |

- **¿El paciente tiene personas dependientes a su cargo (descendientes, cónyuge o ascendientes)?**
- Sí
- No
- **El paciente tiene el apoyo de alguien para que le ayude con su medicación (p.e. cónyuge, familiar, cuidador):**

| - Sí - No | - **¿Tipo de cuidador?** - Formal / remunerado - Informal / no remunerado - **El cuidador tiene algún tipo de vínculo personal o parentesco con el paciente?** - Ninguno - Cónyuge/pareja - Parentesco de primer grado: padres, hijos - Parentesco de segundo grado: hermanos, nietos - Parentesco indirecto: cuñados, yerno/nuera, sobrinos, tíos - Otro_______________________ |
| --- | --- |

1. **Variables sociodemográficas del cuidador**
   1. **Género del cuidador:**

- Hombre
- Mujer
  1. **Edad del cuidador:**

|  | Años |
| --- | --- |

- 1. **Situación laboral del cuidador:**
- **¿El cuidador está activo laboralmente?:**

| - Sí - No | - **¿Qué tipo de jornada laboral realiza?** - Media Jornada - Jornada completa partida - Jornada intensiva (mañana o tarde) - Otra (especificar): _____________________ |
| --- | --- |

- 1. **Estilo de vida del cuidador:**
- **¿Cuál de las siguientes posibilidades describe mejor la frecuencia con la que realiza una actividad física en su tiempo libre?**
- No hago ejercicio. El tiempo libre lo ocupo de forma casi completamente sedentaria (leer, ver la televisión, etc.)
- Hago alguna actividad física o deportiva ocasional (caminar, pasear, ir en bicicleta, jardinería, gimnasia suave, actividades recreativas que requieren un ligero esfuerzo, etc.)
- Hago actividad física varias veces al mes (deportes, gimnasia, correr, natación, ciclismo, juegos de equipo, etc.)
- Hago entrenamiento deportivo o físico varias veces a la semana
- **¿El cuidador tiene otras personas dependientes, a parte del paciente, a su cargo (descendientes, cónyuge o ascendientes)?**
- Sí
- No
- **¿Cuánto tiempo lleva al cuidado del paciente?**

|  | años |  | meses |  |
| --- | --- | --- | --- | --- |

- **¿Al cabo de la semana (lunes a domingo), cuántas horas le dedica al cuidado del paciente aproximadamente?**

|  | Horas |
| --- | --- |

- **¿En qué momentos del día atiende al paciente?**
- Por la mañana (hora del desayuno)
- Al mediodía (hora de la comida)
- Por la tarde (hora de la merienda)
- Por la noche (hora de la cena)
- Durante la noche (después de la cena y hasta la mañana)
  1. **Relación con el paciente**
- **¿Necesita desplazarse para acudir a casa del paciente?**
- Sí, necesito coger el coche o el transporte público
- Sí, puedo ir a pie
- No, vivo con el paciente o en el mismo edificio

1. **Variables clínicas y de tratamiento del paciente**
   1. **Comorbilidades asociadas actuales:**

- Ninguna
- Insuficiencia renal
- Insuficiencia hepática
- Cardiopatía
- Alteraciones gastrointestinales
- Patología neurológica
- Otra (especificar): ___________________________________________
  1. **Riesgo cardiovascular del paciente:**

| - Valor de la escala CHA₂DS₂-VASc |  | - No Disponible |
| --- | --- | --- |
|  |  |  |
| - Valor del índice HAS-BLED |  | - No Disponible |

- 1. **Tratamiento anticoagulante actual:**
- **Tiempo aproximado en tratamiento con ACOD:**

|  | Años |  | Meses |
| --- | --- | --- | --- |

- **ACOD que está recibiendo en este momento**
- Apixaban
- Dabigatran
- Edoxaban
- Rivaroxaban
- **Posología del ACOD que está recibiendo actualmente**

|  | mg |  | veces al día |
| --- | --- | --- | --- |

- **Especialista que le prescribió su tratamiento actual**
- Cardiólogo
- Hematólogo
- Medicina interna
- Neurólogo
- Atención Primaria
- Urgencias
- Otro____________________
- **El ACOD con el que está siendo tratado, ¿es el primer anticoagulante que toma el paciente o había utilizado otro/s antes?**

| - Es el primero - Había usado otro | **¿Cuál tomaba justo antes que el actual?**   - AVK - Otro ACOD (especificar): ______________   **¿Cuál fue el motivo del cambio de tratamiento?**   - Hipersensibilidad al principio activo - Antecedentes de hemorragia intracraneal - Ictus isquémico previo con riesgo de hemorragia intracraneal - Presencia de episodios tromboembólicos - Mal control del INR - Imposibilidad de acceder al control de INR - Otro_______________________________ |
| --- | --- |

- 1. **Medicación concomitante:**
- **En total (incluyendo el anticoagulante que toma)….**
- ¿Cuántos medicamentos toma usted cada día?

| Total diario aproximado: | 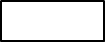 |
| --- | --- |

- ¿En qué momento del día toma alguna medicación?
- Por la mañana
- A mediodía
- Por la noche
- **¿Conoce claramente los medicamentos o alimentos que pueden tener interacción con la medicación anticoagulante que toma?**
- Plenamente
- Conozco algunos
- No los conozco

- 1. **Capacidad de deglución del paciente**
- **¿Tiene el paciente alguna dificultad para tragar la pastilla?**
- Las tengo que triturar siempre
- La tengo que triturar a veces, si son muy grandes
- Las puedo tragar bien

1. **Valoración de atributos específicos del tratamiento con ACOD**
   1. **Importancia relativa respecto a los distintos atributos asociados a los ACOD (SI EL PACIENTE TIENE CUIDADOR O PERSONA QUE LE AYUDE CON LA MEDICACIÓN Y SE ENCUENTRA PRESENTE, AMBOS DEBERÁN RESPONDER A LAS PREGUNTAS DE ESTE APARTADO)**

*Pensando en el tratamiento anticoagulante que toma y asumiendo que todos los ACOD son igual de eficaces en la prevención de eventos cerebrovasculares…*

*Indique, para cada uno de los siguientes atributos, la importancia que el paciente y/o cuidador, a título personal, le otorgaría, basado en su opinión y criterio.).*

| **RESPUESTAS DEL PACIENTE** | | | | | | | | | | | | | | | |
| --- | --- | --- | --- | --- | --- | --- | --- | --- | --- | --- | --- | --- | --- | --- | --- |
|  | Nada de importancia | | | |  | | | | | | | Máxima importancia | | | |
|  |  | 0 | 1 | 2 | | 3 | 4 | 5 | 6 | 7 | 8 | | 9 | 10 |  |
| Aparición de hemorragias graves (que supongan una urgencia hospitalaria) |  | ❑ | ❑ | ❑ | | ❑ | ❑ | ❑ | ❑ | ❑ | ❑ | | ❑ | ❑ |  |
| Aparición de hemorragias leves (gastrointestinal, nasal…) |  | ❑ | ❑ | ❑ | | ❑ | ❑ | ❑ | ❑ | ❑ | ❑ | | ❑ | ❑ |  |
| Posibilidad de interacción con otros medicamentos |  | ❑ | ❑ | ❑ | | ❑ | ❑ | ❑ | ❑ | ❑ | ❑ | | ❑ | ❑ |  |
| Posibilidad de interacción con alimentos |  | ❑ | ❑ | ❑ | | ❑ | ❑ | ❑ | ❑ | ❑ | ❑ | | ❑ | ❑ |  |
| Presencia de lactosa en el medicamento |  | ❑ | ❑ | ❑ | | ❑ | ❑ | ❑ | ❑ | ❑ | ❑ | | ❑ | ❑ |  |
|  |  |  |  |  | |  |  |  |  |  |  | |  |  |  |
| **RESPUESTAS DEL CUIDADOR / PERSONA DE APOYO** | | | | | | | | | | | | | | | |
|  | Nada de importancia | | | |  | | | | | | | Máxima importancia | | | |
|  |  | 0 | 1 | 2 | | 3 | 4 | 5 | 6 | 7 | 8 | | 9 | 10 |  |
| Aparición de hemorragias graves (que supongan una urgencia hospitalaria) |  | ❑ | ❑ | ❑ | | ❑ | ❑ | ❑ | ❑ | ❑ | ❑ | | ❑ | ❑ |  |
| Aparición de hemorragias leves (gastrointestinal, nasal…) |  | ❑ | ❑ | ❑ | | ❑ | ❑ | ❑ | ❑ | ❑ | ❑ | | ❑ | ❑ |  |
| Posibilidad de interacción con otros medicamentos |  | ❑ | ❑ | ❑ | | ❑ | ❑ | ❑ | ❑ | ❑ | ❑ | | ❑ | ❑ |  |
| Posibilidad de interacción con alimentos |  | ❑ | ❑ | ❑ | | ❑ | ❑ | ❑ | ❑ | ❑ | ❑ | | ❑ | ❑ |  |
| Presencia de lactosa en el medicamento |  | ❑ | ❑ | ❑ | | ❑ | ❑ | ❑ | ❑ | ❑ | ❑ | | ❑ | ❑ |  |

- 1. **Posología y vía de administración (SI EL PACIENTE TIENE CUIDADOR O PERSONA QUE LE AYUDE CON LA MEDICACIÓN Y SE ENCUENTRA PRESENTE, AMBOS DEBERÁN RESPONDER A LAS PREGUNTAS DE ESTE APARTADO):**

*Aclaración para el paciente: En este apartado vamos a hablar sobre la forma en la que usted toma la medicación (p.e. oral, inyectada, con comida o sin ella) y la frecuencia con la que lo hace (cuantas veces al día, en qué momento, durante cuánto tiempo, etc).*

- **¿Qué importancia tienen para usted los siguientes aspectos relativos a la medicación que toma?**

| **RESPUESTAS DEL PACIENTE** | | | | | | | | | | | | | | | |
| --- | --- | --- | --- | --- | --- | --- | --- | --- | --- | --- | --- | --- | --- | --- | --- |
|  | Nada de importancia | | | |  | | | | | | | Máxima importancia | | | |
|  |  | 0 | 1 | 2 | | 3 | 4 | 5 | 6 | 7 | 8 | | 9 | 10 |  |
| El número de veces que tiene que tomar el medicamento cada día |  | ❑ | ❑ | ❑ | | ❑ | ❑ | ❑ | ❑ | ❑ | ❑ | | ❑ | ❑ |  |
| El momento en el que tiene que tomar el medicamento (p.e. por la mañana, por la tarde) |  | ❑ | ❑ | ❑ | | ❑ | ❑ | ❑ | ❑ | ❑ | ❑ | | ❑ | ❑ |  |
| Las condiciones que debe cumplir para tomar el medicamento (obligación de tomarlo con alimentos o sin ellos) |  | ❑ | ❑ | ❑ | | ❑ | ❑ | ❑ | ❑ | ❑ | ❑ | | ❑ | ❑ |  |
| La facilidad que ofrece el medicamento para tragarlo (forma y tamaño) |  | ❑ | ❑ | ❑ | | ❑ | ❑ | ❑ | ❑ | ❑ | ❑ | | ❑ | ❑ |  |
| La posibilidad de triturar el medicamento |  | ❑ | ❑ | ❑ | | ❑ | ❑ | ❑ | ❑ | ❑ | ❑ | | ❑ | ❑ |  |
|  |  |  |  |  | |  |  |  |  |  |  | |  |  |  |
| **RESPUESTAS DEL CUIDADOR / PERSONA DE APOYO** | | | | | | | | | | | | | | | |
|  | Nada de importancia | | | |  | | | | | | | Máxima importancia | | | |
|  |  | 0 | 1 | 2 | | 3 | 4 | 5 | 6 | 7 | 8 | | 9 | 10 |  |
| El número de veces que tiene que tomar el medicamento cada día el paciente |  | ❑ | ❑ | ❑ | | ❑ | ❑ | ❑ | ❑ | ❑ | ❑ | | ❑ | ❑ |  |
| El momento en el que tiene que administrar el medicamento al paciente (p.e. por la mañana, por la tarde) |  | ❑ | ❑ | ❑ | | ❑ | ❑ | ❑ | ❑ | ❑ | ❑ | | ❑ | ❑ |  |
| Las condiciones que debe cumplir para administrar el medicamento al paciente (obligación de tomarlo con alimentos o sin ellos) |  | ❑ | ❑ | ❑ | | ❑ | ❑ | ❑ | ❑ | ❑ | ❑ | | ❑ | ❑ |  |
| La facilidad que ofrece el medicamento para tragarlo (forma y tamaño) |  | ❑ | ❑ | ❑ | | ❑ | ❑ | ❑ | ❑ | ❑ | ❑ | | ❑ | ❑ |  |
| La posibilidad de triturar el medicamento |  | ❑ | ❑ | ❑ | | ❑ | ❑ | ❑ | ❑ | ❑ | ❑ | | ❑ | ❑ |  |

1. **Variables sobre preferencias del paciente y/o el cuidador**
   1. **Pensando en el tratamiento anticoagulante oral que toma actualmente… Indique, en una escala del 0 al 10 cómo de SATISFECHO está con el tratamiento anticoagulante que está tomando actualmente**

| **RESPUESTAS DEL PACIENTE** | | | | | | | | | | | | | | |
| --- | --- | --- | --- | --- | --- | --- | --- | --- | --- | --- | --- | --- | --- | --- |
| Nada satisfecho | | |  | | | | | | | | | Máxima satisfacción | | |
|  | 0 | 1 | | 2 | 3 | 4 | 5 | 6 | 7 | 8 | 9 | | 10 |  |
|  | ❑ | ❑ | | ❑ | ❑ | ❑ | ❑ | ❑ | ❑ | ❑ | ❑ | | ❑ |  |

| **RESPUESTAS DEL CUIDADOR** | | | | | | | | | | | | | | |
| --- | --- | --- | --- | --- | --- | --- | --- | --- | --- | --- | --- | --- | --- | --- |
| Nada satisfecho | | |  | | | | | | | | | Máxima satisfacción | | |
|  | 0 | 1 | | 2 | 3 | 4 | 5 | 6 | 7 | 8 | 9 | | 10 |  |
|  | ❑ | ❑ | | ❑ | ❑ | ❑ | ❑ | ❑ | ❑ | ❑ | ❑ | | ❑ |  |

- 1. **Pensando en el tratamiento anticoagulante que toma actualmente, indique un MÁXIMO de 3 factores que considera como MUY POSITIVOS y un máximo de 3 cosas que crea que son MUY NEGATIVAS:**

| **RESPUESTAS DEL PACIENTE** | |
| --- | --- |
| **Muy positivas** | **Muy negativas** |
| 1.  2.  3. | 1.  2.  3. |

| **RESPUESTAS DEL CUIDADOR** | |
| --- | --- |
| **Muy positivas** | **Muy negativas** |
| 1.  2.  3. | 1.  2.  3. |

- 1. **En una situación ideal, si pudiera elegir… ¿Cómo le gustaría tomar su medicación anticoagulante?**

*El paciente o su cuidador debe seleccionar una de las opciones disponibles, la que mejor se adapte a sus necesidades y preferencias*

| **RESPUESTA DEL PACIENTE** | | |
| --- | --- | --- |
| **Grupo A** | **Grupo B** | **Grupo C** |
| - Una toma al día, en cualquier momento con algo de agua | - Una toma al día, siempre acompañada de alimento | - Dos tomas al día (por la mañana y por la noche) |

| **RESPUESTA DEL CUIDADOR** | | |
| --- | --- | --- |
| **Grupo A** | **Grupo B** | **Grupo C** |
| - Una toma al día, en cualquier momento con algo de agua | - Una toma al día, siempre acompañada de alimento | - Dos tomas al día (por la mañana y por la noche) |

**Muchas gracias por su colaboración en el estudio**
